# Supplementary material for: The Construction and Exploration of a Comprehensive MicroRNA Centered Regulatory Network in Foxtail Millet (Setaria italica L.)
Source: Front Plant Sci. 2022 May 6;13:848474. doi: 10.3389/fpls.2022.848474 (PMC9121102; doi:10.3389/fpls.2022.848474)
Supplement: Supplementary file 7 [file Data_Sheet_7.zip › Sit-miRN2548_Seita.4G139900.1_780_TPlot.pdf]

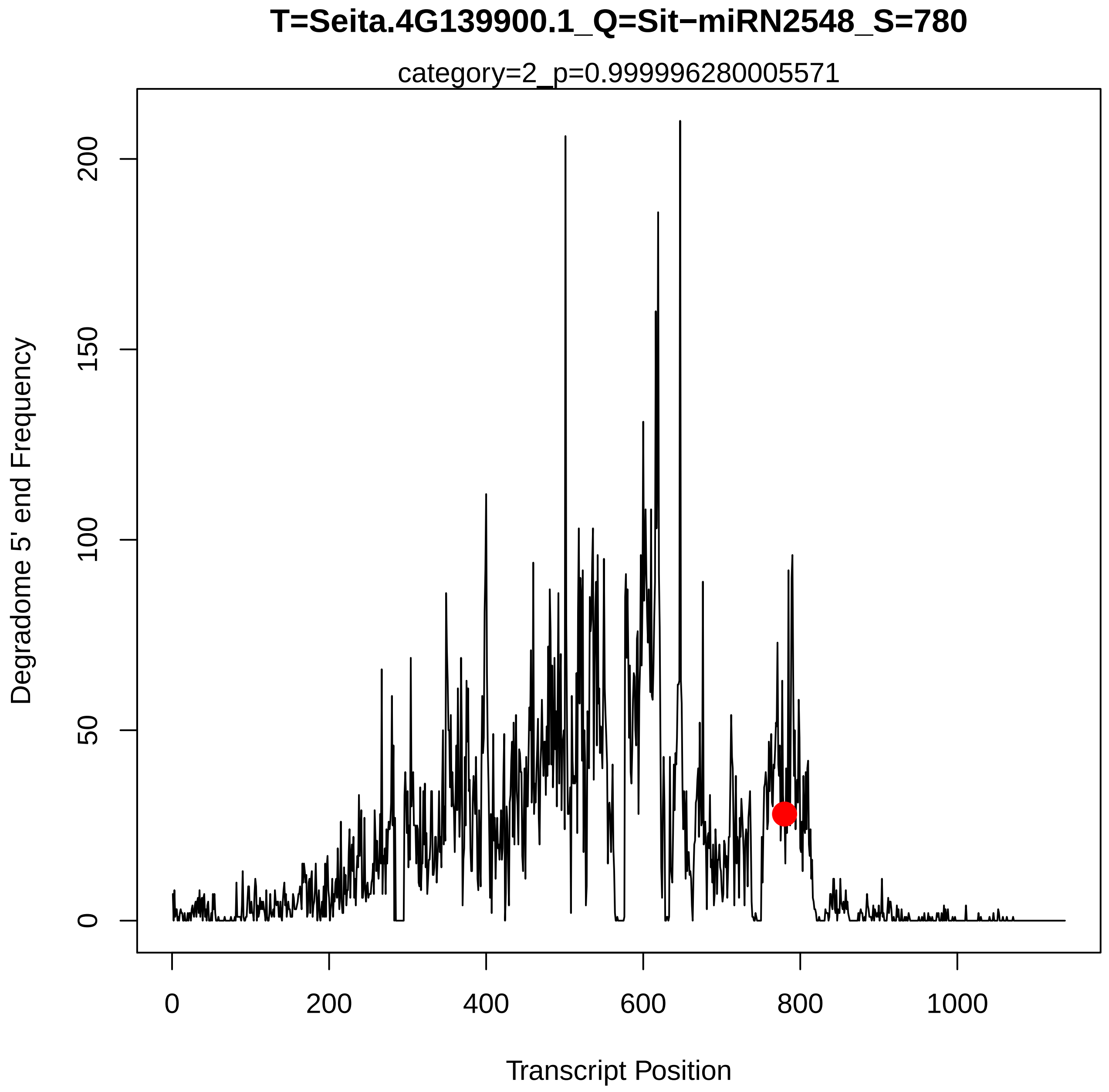
T=Seita.4G139900.1_Q=Sit−miRN2548_S=780
category=2_p=0.999996280005571
Degradome 5' end Frequency
0
50
100
150
200
0
200
400
600
800
1000
Transcript Position
